# A Scoping Review of Innovative Housing for Older Adults: Focus on Homesharing, Cohousing, NORC-SSPs, and Villages

**DOI:** 10.1093/geroni/igaa057.102

**Published:** 2020-12-16

**Authors:** Kishore Seetharaman, Atiya Mahmood, Habib Chaudhury

**Affiliations:** Simon Fraser University, Vancouver, British Columbia, Canada

## Abstract

This scoping review explores the benefits, and challenges of major innovative housing and service models for older adults. Several countries are prioritizing housing and service innovation for older adults to address the limitations of traditional market housing in meeting their housing needs and challenges. This study contributes to this issue by reviewing peer-reviewed and grey literature on four well-established innovative housing and service models: cohousing, homesharing, Naturally Occurring Retirement Community Supportive Services Programs (NORC-SSPs), and Villages. Based on the findings synthesized from 65 sources, a pair-wise comparison of (i) cohousing and homesharing; and (ii) NORC-SSPs and Villages was conducted on the basis of: (a) financial aspects (e.g., affordability); and (b) psychosocial aspects (e.g., social interaction and engagement, intergenerationality, autonomy and interdependence, safety, diversity and inclusivity); and (c) long-term sustainability. While there were several financial and psychosocial benefits related to each model, the following challenges were identified that need to be addressed to further improve the relevance and effectiveness of these models: (i) lack of funding and resources to support NORC-SSPs and Villages; (ii) inter-resident conflict in homesharing and cohousing; (iii) limitations of informal support provided by fellow-residents in meeting the needs of older adults with complex needs in all four models; and (iv) lack of inclusivity and sociocultural diversity in cohousing and Villages. By integrating research on older adults’ housing needs and innovative solutions, the findings of this study could guide future housing initiatives that seek to adopt these innovative models by highlighting their strengths, while recognizing areas for improvement.

